# Risk factors for skip metastasis in patients with papillary thyroid microcarcinoma

**DOI:** 10.1002/cam4.5507

**Published:** 2022-12-08

**Authors:** Xin Wu, Binglu Li, Chaoji Zheng, Xiaodong He

**Affiliations:** ^1^ Department of General Surgery Peking Union Medical College Hospital, Chinese Academy of Medical Sciences and Peking Union Medical College Beijing China

**Keywords:** lymph node metastasis, papillary thyroid microcarcinoma, risk factor, skip metastasis

## Abstract

**Background:**

Lymph node metastasis (LNM) is prevalent in papillary thyroid microcarcinoma (PTMC) and is essential when determining tumor stage and prognosis. Positive lateral LNM with negative central LNM is defined as skip metastasis. Thyroid carcinoma's risk factors for skip metastasis remain controversial, especially in PTMC. This study aimed to determine the clinical features as well as the risk factors of skip metastasis among patients with PTMC.

**Methods:**

We conducted retrospective research among patients with PTMC who were subjected to treatment at our Hospital between January 2018 and December 2019 by reviewing their medical records. A database containing demographic characteristics, ultrasonography features, blood test outcomes, operation information, pathology details, and follow‐up information was constructed. The link between skip metastasis and clinicopathological features of PTMC was evaluated using univariate as well as multivariate analyses.

**Results:**

Overall, 293 patients diagnosed with PTMC and lateral LNM were included. There were 91 men (31.1%) and 202 women (68.9%). The median age was 38 (31–47) years. Fifty patients were diagnosed with skip metastases. Levels III and II + III were the most prevalent in single‐level and two‐level metastasis, correspondingly. Univariate and multivariate analyses detected two independent factors linked to skip metastasis in PTMC: female sex (odds ratio = 2.609, 95% confidence interval (CI): 1.135–6.000; *p* = 0.024) and location of the tumor (upper portion) (odds ratio = 2.959, 95% CI: 1.552–5.639; *p* = 0.001).

**Conclusions:**

Skip metastasis is prevalent in thyroid carcinoma. Female sex and tumor location (upper portion) are independently linked to skip metastasis in PTMC. Patients who have these two risk factors should undergo a meticulous preoperative and intraoperative evaluation of lymph node status.

## Graphical abstract

Skip metastasis is not uncommon in thyroid carcinoma. This study analyzed the risk factors for skip metastasis among patients with papillary thyroid microcarcinoma. The incidence was 1.1%, and female sex and tumor location (upper portion) were independently linked to skip metastasis. As such, we propose that patients who had these two risk factors should be subjected to meticulous preoperative and intraoperative evaluation of lymph node status.

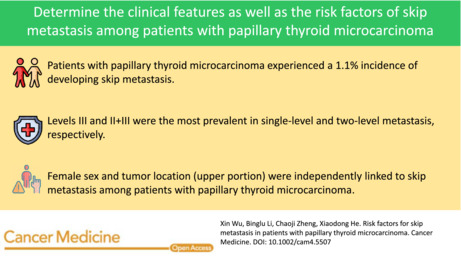

## 1 INTRODUCTION

Thyroid carcinoma incidence has risen dramatically over the past decades.^1^, ^2^, ^3^ In China, there are more than 90,000 new thyroid cancer cases each year.^4^ In South Korea, thyroid cancer has become the most commonly diagnosed tumor among women.^2^ Between 1975 and 2009, the yearly incidence of thyroid carcinoma in the United States approximately tripled.^5^ Lymph node metastasis (LNM) is prevalent in thyroid cancer and can be classified into central and lateral compartment metastases. The former includes pretracheal, paratracheal, and prelaryngeal (Delphian) lymph nodes, and the latter includes levels II, III, IV, and V. Generally, ipsilateral central LNM is the first step of LNM, followed by contralateral central LNM and lateral LNM.^6^ However, lateral LNM may occur in a negative central lymph node. This situation is defined as skip metastasis and its incidence is reportedly 1.6%–21.8% of papillary thyroid carcinoma.^7^, ^8^, ^9^

The rising incidence of thyroid carcinoma is concomitant with an increase in low‐risk, indolent, and small papillary thyroid cancers.^10^ Papillary thyroid microcarcinoma (PTMC) refers to a papillary thyroid carcinoma with a tumor diameter ≤ 10 mm. Several previous studies have reported different predisposing factors for skip metastasis in papillary thyroid carcinoma, and some studies revealed that PTMC might be associated with skip metastasis.^8^, ^9^, ^11^ However, no study has reported the predisposing factors and predictors of skip metastasis in PTMC. Skip metastatic lymph nodes are easily missed during preoperative examination and surgery. LNM can affect local recurrence and long‐term survival.^12^ If skip metastasis is missed and untreated, it will increase the recurrence rate, deteriorate patients' quality of life, and make the prognosis worse.^13^ As a result, it is crucial to recognize the risk factors of skip metastasis, which can improve the treatment effect of selected patients. Our aim was to explore the incidence, clinical features, and predisposing factors of skip metastasis in PTMC and to improve the clinical management of PTMC.

## 2 MATERIAL AND METHODS

The ethical approval for conducting this experiment was granted by the Peking Union Medical College Hospital's Institutional Review Board (I‐22PJ053). Because of the retrospective nature of the research, informed consent for data publishing was not required.

### 2.1 Patients

We conducted retrospective research among patients with PTMC who were subjected to treatment at Peking Union Medical College Hospital between January 2018 and December 2019 by reviewing their medical records. Patients' recruitment into the study was done on the basis of the following inclusion criteria: (1) PTMC was diagnosed by postoperative pathology; (2) lateral LNM confirmed via pathological paraffin section; (3) complete medical records. On the other hand, the exclusion criteria were as follows: (1) reoperation, (2) papillary thyroid carcinoma mixed with follicular/medullary/anaplastic cancer, (3) distant metastasis, (4) head and neck radiation history, and (5) non‐curative surgery. Both inpatient and outpatient medical data records were reviewed, and clinical data were recorded and integrated by two independent individuals. A retrospective database with demographic characteristics, ultrasonography features, blood test results, operation data, pathology details, and follow‐up data was set up for investigation.

### 2.2 Treatment

All the patients were investigated by thyroid function tests as well as ultrasonography before surgery. The operation was conducted under general anesthesia with no use of antibiotics. The diagnosis of thyroid carcinoma was done by preoperative fine‐needle aspiration biopsy or intraoperative freezing and subjected to confirmation by postoperative pathology. The total thyroidectomy with central and lateral lymph node dissection was conducted for all the patients. The degree of central lymph node dissection incorporated levels VI (pretracheal, paratracheal, and prelaryngeal/Delphian), and the extent of lateral lymph node dissection included levels II, III, IV, and V (high, mid‐, and lower jugular and supraclavicular). All patients received euthyrox suppression therapy after surgery, and the thyroid‐stimulating hormone level was sustained at around 0.1 μIU/mL. During the first year after surgery, all patients were followed up every 3 months, and then every 6 months after that. Thyroid function tests, ultrasonography, radiography, and chest computed tomography were used to assess the patient's condition.

### 2.3 Definition

A tumor was defined as a multifocal lesion when a minimum of two foci were discovered in the unilateral or bilateral lobes. The major tumor was a tumor with the maximum diameter of the lesion, whereas the total tumor sizes referred to the sum of the diameters of all lesions. The upper, middle, and lower portions of the tumor location were defined as the upper of the high plane of the isthmus, parallel to the isthmus, and lower of the low plane of the isthmus, respectively. The normal reference range for thyroid‐stimulating hormone is 0.38–4.34 μIU/mL.

### 2.4 Statistical analysis

The SPSS software (version: 25.0; IBM Corp.) was utilized for all analyses. Categoric variables were presented as absolute numbers or frequencies and continuous variables were shown as the median (25th–75th percentiles). Differences between study groups were compared and investigated by Chi‐square test or Fisher's exact test, as applicable. The independent risk factors for skip metastases in PTMC were identified using logistic multivariate regression analysis. The threshold for statistical significance was fixed at *p* < 0.05.

## 3 RESULTS

From January 2018 and December 2019, 4457 individuals with PTMC were treated at the Peking Union Medical College Hospital. Lateral lymph node dissection was performed for 432 patients, and 293 patients with positive lateral LNM were enrolled in the present study. There were 91 men (31.1%) and 202 women (68.9%), with a male‐to‐female ratio of 1:2.2. The median age was 38 (31–47) years. At our hospital, all of the patients had primary radical surgery. Only 26 individuals had bilateral lateral lymph node dissection, whereas the other 267 had unilateral lateral lymph node dissection. Major and total tumor lesions had median diameters of 0.7 (0.5–0.9) cm and 1.0 (0.7–1.2) cm, correspondingly. The median number of metastatic and harvested lymph nodes in the central compartment was 3 (1–6) and 9 (6–14), respectively. The median number of metastatic and harvested lymph nodes in the lateral compartment was 3 (2–5) and 20 (15–28), respectively.

On the basis of postoperative pathology, patients were classified into two distinct groups, namely, the skip metastasis (lateral LNM without central LNM) group (*n* = 50) and the normal (lateral LNM with central LNM) group (*n* = 243). The skip metastasis incidence among patients with PTMC was 1.1% (50/4457). The detailed skip metastasis distribution according to neck level is shown in Table 1. Levels III and II + III were the most prevalent in single‐level and two‐level metastasis, respectively. The demographic data, laboratory assay results, and sonographic features were presented and subjected to a comparison between the groups (Table 2). The skip metastasis group had significantly more female patients than the normal group. The pathology results of the two groups were analyzed and compared (Table 3). Compared with the normal group, the skip metastasis group demonstrated fewer patients with extrathyroidal invasion and a great number of patients with non‐classical subtype and upper portion lesions. Multivariate logistic regression analyses were utilized to determine the association between skip metastasis and select variables as demonstrated in Table 4. Two variables were independently linked to skip metastasis: female sex (odds ratio [OR] = 2.609, 95% confidence interval [CI]: 1.135–6.000; *p* = 0.024) and tumor location (upper portion) (OR = 2.959, 95% CI: 1.552–5.639; *p* = 0.001).

**TABLE 1:** The distributions of skip metastasis according to neck level

Distribution	Patients number
Single level	24
Level III	16
Level IV	5
Level II	3
Two levels	22
Level II, III	12
Level III, IV	7
Level II, IV	2
Level III, V	1
Three levels	3
Level II–IV	3
Four levels	1
Level II–V	1

**TABLE 2:** Comparison of demographic data and test results between two groups of patients with PTMC

Factors	Skip metastasis group (*n* = 50)	Normal group (*n* = 243)	*p*
Sex (male/female)	8/42	83/160	0.012
Age ≥ 55 years	6	28	0.924
BMI ≥ 28 kg/m^2^	3	37	0.084
Thyroid cancer family history	3	8	0.611
Smoking	4	30	0.382
Hypertension	5	18	0.740
Diabetes	3	10	0.832
Hyperthyroidism	0	3	1.000
TSH ≥2.5 μIU/mL	11	64	0.522
Hashimoto's disease	9	55	0.470
Taller than wide	37	183	0.846
Irregular shape	10	31	0.179
Calcification	38	177	0.645
Affluent blood flow	8	55	0.298
Hypoecho	47	232	0.936

Abbreviations: BMI, body mass index; PTMC, papillary thyroid microcarcinoma; TSH, thyroid‐stimulating hormone.

**TABLE 3:** Comparison of pathology results between two groups of patients with PTMC

Factors	Skip metastasis group (*n* = 50)	Normal group (*n* = 243)	*p*
Tumor location (left/right)	22/28	113/130	0.747
Tumor subtype (classical/non‐classical)	36/14	210/33	0.011
Multifocality	21	125	0.224
Bilaterality	16	82	0.812
Capsule invasion	37	189	0.562
Extrathyroidal invasion	4	51	0.032
Cell proliferation antigen Ki 67 ≥ 3%	6	47	0.219
Major tumor size			
≤0.5 cm	13	61	0.894
>0.5 cm	37	182	
Total tumor size			
≤0.5 cm	10	37	0.467
0.5 ~ 1 cm	27	123	
>1 cm	13	83	
Tumor location (upper/middle/lower)	29/17/4	82/131/30	0.006
Tumor location (upper/middle + lower)	29/21	82/161	0.001
No. of harvested LNs in CC	6.5 (3–11)	10 (6–15)	‐‐
No. of metastatic LNs in CC	0	4 (2–7)	‐‐
No. of harvested LNs in LC	21 (17–26.5)	20 (15–28)	‐‐
No. of metastatic LNs in LC	2.5 (1–4)	3 (2–5)	‐‐

Abbreviations: CC, central compartment; LC, lateral compartment; LNs, lymph nodes; PTMC, papillary thyroid microcarcinoma.

**TABLE 4:** Multivariate analysis of risk factors for skip metastasis among PTMC patients

	*p*	OR	95% CI
Tumor location (upper portion)	0.001	2.959	1.552–5.639
Female sex	0.024	2.609	1.135–6.000
No extrathyroidal invasion	0.056	2.912	0.975–8.699
Non‐classical subtype	0.073	2.010	0.937–4.313

Abbreviations: CI, confidence interval; PTMC, papillary thyroid microcarcinoma; OR, odds ratio.

All patients had been followed up as of October 2021, with a median follow‐up duration of 34 (28–39) months. In addition to thyroid function tests, neck ultrasonography and chest computed tomography were used to assess the patient's condition. No local recurrence or distant metastases were detected.

## 4 DISCUSSION

Skip metastasis is not uncommon in thyroid carcinoma, but there have only been a few studies published on this subject, especially among individuals with PTMC. This study analyzed the risk factors for skip metastasis among PTMC patients based on a large set of patient data. Patients with PTMC experienced a 1.1% incidence of developing skip metastases, according to our findings. Also, female sex and tumor location (upper portion) were independently linked to skip metastasis among PTMC patients. As such, we propose that PTMC patients who had these two risk factors should be subjected to meticulous preoperative and intraoperative evaluation of lymph node status.

According to the American Joint Committee on Cancer (AJCC) Cancer Staging Manual (8th edition),^14^ lymph node status is very important in tumor staging, especially in patients aged ≥55 years. LNM is very common in thyroid cancers and several previous studies have reported that LNM was found in about 30–80% of patients with papillary thyroid carcinoma.^15^, ^16^, ^17^ Accurate assessment of lymph node status is crucial because of the importance and prevalence of LNM. Skip metastasis is one of the most neglected aspects of LNM. Therefore, strengthening the research on skip metastasis is helpful in improving the therapeutic management of thyroid carcinoma. A previous systematic review and meta‐analysis study, which included 18 research reports with a sample size of 2165 patients, indicated that the upper pole location and a tumor size ≤1 cm were significantly associated with skip metastasis.^6^ The authors also analyzed several other variables, such as sex, age, multifocality, bilaterality, extrathyroidal extension, and thyroiditis, but found no significant relationship between them and skip metastasis.^6^ In the present study, we focused on PTMC and identified two associated risk factors: female sex and tumor location (upper portion).

Several previous studies have reported tumor location as an independent risk factor for skip metastasis. Wang et al.^13^ studied 378 participants with papillary thyroid carcinoma and found that a primary tumor location in the upper portion, a primary tumor size of ≤1 cm, and age were independently associated with skip metastasis. Feng et al.^18^ also revealed that tumors in the upper lateral pole and a tumor size ≤1 cm were independent risk factors for skip metastasis. Our findings were in line with those of prior studies and confirmed the significance of tumor location in skip metastasis in patients with PTMC. Different thyroid locations may have different lymphatic drainage patterns. Lymphatic drainage along the inferior thyroid vein facilitates the transportation of cancerous cells from the lower compartment to the central compartment. Meanwhile, the lymphatic flow of the upper portion is along the superior thyroid vessels, so cancerous cells from the upper portion are transported easily to the lateral compartment.^19^, ^20^ This possible lymphatic pathway was confirmed by Likhterov et al. through lymphatic anatomic studies.^21^ This might explain why a tumor located in the upper portion increases the risk of skip metastasis. But this exclusive way of lymphatic drainage which bypasses the central compartment may not be present in all thyroid carcinomas, otherwise skip metastasis would be a very common phenomenon. More anatomical and physiological studies are needed to confirm whether PTMC has other unique lymphatic pathways. Besides tumor location, female sex was identified to be a risk factor for skip metastasis among PTMC patients. The role of sex in LNM in thyroid carcinoma is controversial. Numerous previous studies have suggested a link between LNM and sex in PTMC; male sex was found to be a predisposing factor for LNM in patients with PTMC.^22^, ^23^, ^24^ However, many other studies found no relationship between LNM and sex.^25^, ^26^ The physiological mechanism by which sex affects LNM remains unclear. One possible explanation is that the pituitary gonadotropin‐releasing hormone levels in women can be affected by higher levels of estrogen and progesterone.^27^, ^28^ At the same time, men have a higher basal metabolic rate than women, which may lead to an overactive proliferation of tumor cells. To better understand the function of sex in LNM and skip metastases, more studies are needed.

Several other factors have been linked to skip metastasis in papillary thyroid carcinoma. Age has been identified as an independent risk factor for skip metastasis among individuals with papillary thyroid carcinoma.^13^, ^29^, ^30^, ^31^ Different studies used watersheds of different ages, with Dou et al. suggesting an age of 55 years^29^, ^30^ and Hou J suggesting 44.5 years.^31^ However, the general trend was that older patients are at a high risk of developing skip metastasis. Jin et al.^11^ studied 355 patients with papillary thyroid carcinoma and found that skip metastasis was commonly detected among patients with extrathyroidal extension. Hu et al.^30^ analyzed 745 patients and demonstrated that unilaterality was associated with skip metastasis. Huang et al.^32^ reported 304 patients with papillary thyroid carcinoma with lateral LNM and revealed that in skip metastasis, preoperative serum thyroglobulin served as a risk factor. This study found no remarkable link between these factors and skip metastasis. The possible reasons were that only patients with PTMC were included in this research and the number of patients included was limited.

Although it remains unstandardized, the main treatment of PTMC is surgery.^33^, ^34^, ^35^ The controversy is the extent of operation. Hemithyroidectomy is appropriate for most PTMC patients, and total thyroidectomy should be discussed preoperatively and performed in patients with specific features such as advanced age, extrathyroidal spread, multifocality, and distant metastases. Meanwhile, for both central and lateral lymph nodes, therapeutic dissection is recommended in patients with PTMC, but prophylactic dissection is not.^34^, ^35^ Therefore, it is very important to accurately assess the status of lymph nodes. Both preoperative ultrasonography and intraoperative exploration are commonly used evaluation methods. But they may fail to detect positive lymph nodes, which may result in some patients who should undergo lymph node dissection not receiving this operation.^15^ Summarizing the risk factors of LNM is beneficial to selectively perform more rigorous preoperative examination, intraoperative exploration and necessary lymph node biopsy in high‐risk patients. Compared with the central compartment, the lateral compartment is more likely to be neglected preoperatively and intraoperatively because it is not adjacent to the thyroid and belongs to the second station of LNM. For patients with high risk of skip metastasis, even if the central compartment is negative, rigorous preoperative evaluation and intraoperative examination of the lateral compartment are very important to avoid missing potentially positive lymph nodes.

To the best of our knowledge, almost all previous studies on skip metastasis have focused on papillary thyroid carcinoma. Our study is one of the first studies to focus on patients with PTMC. Meanwhile, the count of harvested lymph nodes in both the central and lateral compartments was sufficient in our study. However, this research has some drawbacks. Firstly, the registration data and investigated variables could not be specified in advance owing to their retrospective nature. Secondly, the number of included patients was limited due to the single‐center feature and the low skip metastasis incidence among patients with PTMC. Prospective, observational, and multicenter clinical experiments are required to derive more supporting evidence with higher reliability.

In conclusion, our results suggest that female sex and tumor location (upper portion) are independent factors for skip metastasis among patients with PTMC. This is one of the first studies of skip metastasis in PTMC. This will make people's understanding of PTMC more comprehensive. Therefore, patients with these two factors should undergo a meticulous preoperative and intraoperative evaluation of lymph node status. These measures will play a positive role in reducing the recurrence rate and improving the long‐term prognosis of PTMC.

## AUTHOR CONTRIBUTIONS

All authors contributed to this manuscript: Xin Wu and Binglu Li prepared the conception and design of this research as well as data analysis, Xin Wu and Chaoji Zheng contributed to drafting the manuscript, Xin Wu and Xiaodong He conducted the statistical analysis and report within the manuscript. Binglu Li revised the final article. All authors read and approved this manuscript and declare they have no conflict of interest.

## FUNDING INFORMATION

The National High Level Hospital Clinical Research Funding (2022‐PUMCH‐A‐054) provided funds for this research.

## CONFLICT OF INTEREST

No potential conflict of interest concerning this study was reported.

## ETHICAL APPROVAL

The ethical approval for conducting this experiment was granted by the Peking Union Medical College Hospital's Institutional Review Board (I‐22PJ053). Due to the retrospective nature of the research, informed consent for data publishing was not required.

## Data Availability

There is no extra information available.
